# Pre- and Post-Operative Cognitive Assessment in Patients Undergoing Surgical Aortic Valve Replacement: Insights from the PEARL Project

**DOI:** 10.3390/neurosci5040035

**Published:** 2024-10-28

**Authors:** Valentina Fiolo, Enrico Giuseppe Bertoldo, Silvana Pagliuca, Sara Boveri, Sara Pugliese, Martina Anguissola, Francesca Gelpi, Beatrice Cairo, Vlasta Bari, Alberto Porta, Edward Callus

**Affiliations:** 1 Clinical Psychology Service, IRCCS Policlinico San Donato, San Donato Milanese, 20097 Milan, Italy; valentina.fiolo@grupposandonato.it (V.F.); silvana.pagliuca@grupposandonato.it (S.P.); edward.callus@grupposandonato.it (E.C.); 2 Laboratory of Biostatistics and Data Management, Scientific Directorate, IRCCS Policlinico San Donato, 20097 Milan, Italy; sara.boveri@grupposandonato.it; 3 Department of Cardiothoracic, Vascular Anesthesia and Intensive Care, IRCCS Policlinico San Donato, San Donato Milanese, 20097 Milan, Italy; sarapugliese96@icloud.com (S.P.); martina.anguissola@grupposandonato.it (M.A.); vlasta.bari@grupposandonato.it (V.B.); alberto.porta@unimi.it (A.P.); 4 Department of Biomedical Sciences for Health, University of Milan, 20133 Milan, Italy; francesca.gelpi@grupposandonato.it (F.G.); beatrice.cairo@unimi.it (B.C.)

**Keywords:** aortic valve stenosis (AVS), surgical aortic valve replacement (SAVR), neurocognitive impairment, cognitive outcomes, verbal episodic memory, executive functions, neuropsychological assessment, cognitive stability, postoperative cognitive improvement, visuospatial abilities

## Abstract

Background: Aortic valve stenosis (AVS) is a common valvular heart disease affecting millions of people worldwide. It leads to significant neurocognitive and neuropsychological impairments, impacting patients’ quality of life. Objective: The objective of this article is to identify and discuss the potential neurocognitive effects on patients with aortic stenosis before and after undergoing surgical aortic valve replacement (SAVR). Method: Our study involved the assessment of 64 patients undergoing aortic valve replacement (SAVR) using a neurocognitive evaluation comprising a battery of 11 different cognitive tests. These tests were designed to analyze the patients’ overall cognitive functioning, executive abilities, short- and long-term memory, and attentional performance. The tests were administered to patients before the aortic valve surgery (T0) and after the surgery (T1). From a statistical perspective, numerical variables are presented as means (±standard deviation) and medians (IQR), while categorical variables are presented as counts and percentages. Normality was assessed using the Shapiro–Wilk test. T0 and T1 scores were compared with the Wilcoxon signed rank test, with *p* < 0.05 considered significant. Analyses were performed using SAS version 9.4. Results: Conducted as part of a fully financed Italian Ministry of Health project (RF-2016-02361069), the study found that most patients showed normal cognitive functioning at baseline. Cognitive assessments showed that executive functions, attention, language, and semantic knowledge were within the normal range for the majority of participants. After SAVR, cognitive outcomes remained stable or improved, particularly in executive functions and language. Notably, verbal episodic memory demonstrated significant improvement, with the percentage of patients scoring within the normal range on the BSRT increasing from 73.4% at T0 to 92.2% at T1 (*p* < 0.0001). However, visuospatial and visuoconstructive abilities showed stability or slight decline, while attentional skills remained relatively stable. The Clock Drawing Test indicated the maintenance of cognitive functions. Conclusions: The findings of our study indicate a global stability in cognitive status among patients after undergoing SAVR, with significant improvement noted in verbal episodic memory. While other cognitive domains did not demonstrate statistically significant changes, these insights are valuable for understanding the cognitive effects of SAVR and can guide future research and clinical practice in selecting the most effective surgical and rehabilitative options for patients. Monitoring cognitive outcomes in patients undergoing aortic valve replacement surgery remains crucial.

## 1. Introduction

Aortic valve stenosis (AVS) is a progressive disorder that narrows the aortic valve, leading to symptoms like angina, syncope, and dyspnea. Left untreated, it can be fatal [1]. In Europe and North America, it is the most common valvular pathology requiring cardiac surgery intervention, and its incidence is rising due to the aging population [2]. AVS is caused primarily by valve calcification, congenital defects, or rheumatic heart disease. As the prevalence of aortic stenosis increases with age, it poses significant challenges to healthcare systems, society, and the economy. Therefore, the objective of this study is to examine whether the surgical aortic valve replacement (SAVR) methodology is associated with cognitive outcomes by conducting neuropsychological assessments before (T0) and after surgery (T1).

## 2. AVS and Cognitive Function: Impact on Neurocognition

AVS not only affects the cardiovascular system, but can also result in neurocognitive issues due to reduced cerebral blood flow. Patients may experience dizziness, confusion, and memory problems, and in severe cases, it can lead to strokes and cognitive decline [3]. While no medical therapy has proven to be effective, SAVR and transcatheter aortic valve implantation (TAVI) are common treatments [2]. TAVI, a less-invasive alternative, has shown promising outcomes, but requires further examination of its impact on neurocognition, especially in older adults [4]. Some studies suggest that TAVI may have advantages over SAVR in terms of stroke incidence and recovery time [5], but more research is needed to fully understand their effects on neurocognitive outcomes.

## 3. Aging of Population in Italy

The aging population of Italy has been a concern for many years, influenced by both endogenous factors such as increased life expectancy and decreased fertility, as well as exogenous factors such as migration flows. This demographic shift has significant implications for healthcare. The prevalence of AVS, the most common valvular heart disease in the elderly, is increasing [6].

Life expectancy has risen, and researchers have proposed a dynamic measure to calculate the third-age threshold, starting at around 73 years for men and 76 years for women. This offers a different perspective on population aging and a better assessment of the social and economic impact.

However, despite increased life expectancy, health conditions do not decline gradually. Morbidity is contracting, but this could pose challenges in the management of aortic valve stenosis, as increased morbidity and comorbidity may limit therapeutic options for patients and cardiologists.

Studies have suggested a possible association between aortic stenosis (AS) and cognitive decline in older adults. Cardiac surgery, both SAVR and TAVI, has been linked to cognitive decline, with some reporting an increased incidence of postoperative cognitive dysfunction (POCD).

Further research is needed to understand the underlying mechanisms of cognitive decline in patients with AVS undergoing cardiac surgery. Long-term assessments of the impact of SAVR and TAVI on neuropsychological and neurocognitive functioning are crucial.

## 4. Background of Our Study

Numerous studies have explored the association between heart health and brain health, indicating a strong link between the two [7]. The brain’s high energy demand, accounting for 20% of the body’s oxygen consumption despite being only 2% of total body weight, underscores its vulnerability to cardiovascular issues. Cerebrovascular alterations such as hemorrhagic infarctions, ischemic cortical infarctions, vasculopathies, and white matter changes could increase the risk of dementia. In fact, heart attacks can damage critical brain regions, particularly those associated with memory, such as the thalamus and thalamo-cortical projections, further emphasizing the brain’s dependence on a healthy cardiovascular system for optimal function [7].

Vascular pathology has been shown to contribute to chronic cerebral hypoperfusion, blood–brain barrier rupture, and inflammation, potentially leading to neuronal death and neurodegeneration [8]. Research has also linked heart diseases to cognitive impairment, with proposed mechanisms like brain tissue hypoperfusion and atrophy in critical areas [9,10].

The assessment of cognitive function in patients undergoing SAVR and TAVI procedures has been conducted in situations of chronic hypoperfusion. The selected articles, including reviews and experimental trials, examined changes in cognitive functions before and after SAVR or TAVI, and some compared the two procedures.

It is evident that SAVR and TAVI are associated with a risk of neuropsychological dysfunction, such as cognitive decline, stroke, and delirium, mainly attributed to embolic material dislodgment during the procedures, affecting brain function [11]. However, the incidence and severity of these dysfunctions vary across studies, and contributing factors are not fully understood.

It is crucial to consider that patients with aortic valve stenosis may experience various neurocognitive and neuropsychological issues before surgery. Studies have shown lower cognitive test scores in patients with aortic valve stenosis compared to age-matched controls, and the severity of cognitive impairment correlates with the severity of the valve condition [11]. Reduced cerebral blood flow and oxygenation have also been observed in these patients, further supporting the role of cerebral hypoperfusion in cognitive decline.

In summary, the literature shows a significant connection between heart and brain health. While SAVR and TAVI are associated with neuropsychological risks, further research is needed to fully comprehend the underlying mechanisms and potential differences between the two procedures. Understanding this complex interaction is crucial for optimizing patient outcomes and quality of life.

## 5. SAVR and Impact on Neurocognitive Outcomes

Several studies have investigated the relationship between SAVR and neurocognitive outcomes.

A study by Spaziano et al. (2014) [12], which examined the neurological outcomes of patients undergoing surgical aortic valve replacement (SAVR), used diffusion-weighted MRI (DW-MRI) to assess the presence of new cerebral lesions and their impact on cognitive function. The study found that a significant proportion of SAVR patients developed new DW-MRI lesions, although the clinical stroke rate remained low, ranging from 0% to 4.8%. These findings suggest that while SAVR is associated with new cerebral lesions, these lesions do not appear to have a lasting impact on cognitive function or correlate with clinical stroke events.

Selnes et al. [13] found that 27% of patients experienced cognitive decline one month after SAVR, particularly in those with pre-existing cognitive impairment. Newman et al. [14] revealed that 12% of patients developed new neurocognitive deficits one week after the surgery, with more significant in patients with longer cross-clamp times.

More pronounced deficits were observed in patients with pre-existing cognitive impairment and longer bypass times. De Rui et al. [15] observed stable cognitive scores at 3 and 6 months but a significant decrease at 12 months compared to baseline.

Recent trials did not specifically examine the cognitive profile of SAVR patients. However, other studies [16] have highlighted that while minimal or insignificant cognitive changes were observed before and after surgery in some cases, Giovannetti et al. [16] emphasize a different aspect. Their findings indicate that patients undergoing AVR had lower scores on tests of working memory and inhibition at 4–6 weeks post-surgery compared to older adults with similar cardiovascular disease who did not undergo surgery. Additionally, post-operative cognitive dysfunction at 4–6 weeks is associated with a higher number and larger size of acute cerebral infarcts.

## 6. Material and Methods

The objective of this study is to examine whether SAVR is associated with cognitive outcomes by conducting neuropsychological assessments. Specifically, our goals are as follows:

1. Assess the neurocognitive status of 104 patients undergoing SAVR using a comprehensive battery of neuropsychological and cognitive tests before surgery (T0) and after surgery (T1). This evaluation aims to characterize the neurocognitive functions of patients both before the SAVR procedure and within seven days post-intervention.
2. Determine through statistical analysis of indices that describe specific cognitive domains whether a relationship exists between surgical aortic valve replacement and the cognitive profile of patients, and how this relationship evolves during follow-up.
3. To evaluate the cognitive state of the patients, the following neurocognitive tests were utilized due to their standardization across diverse populations and proven effectiveness in measuring various aspects of cognition:
  1. Mini Mental State Examination (MMSE) [17].
  2. Digit Span (DST) [18].
  3. Corsi Block Tapping Test (CBTT) [18].
  4. Babcock Story Recall Test (BSRT) [19].
  5. Phonemic Verbal Fluency (PVF) [19].
  6. Semantic Verbal Fluency (SVF) [19].
  7. Denomination and Objects Detection Test (DODT) [20].
  8. Segments Discrimination Test (SDT) [21].
  9. Clock Drawing Test (CDT) [22].
  10. Attentional Matrices Test (AMT) [21].
  11. Frontal Assessment Battery (FAB) [23].

The MMSE is a widely used screening tool for assessing cognitive impairment. It consists of a series of questions and tasks that evaluate various cognitive domains, including orientation, memory, attention, language, and visuospatial abilities. The score obtained from this examination helps determine the severity of cognitive impairment [17].

The DST measures working memory and attention. Participants are required to repeat a sequence of digits in the same order (forward span) or in reverse order (backward span). This test assesses the individual’s ability to remember and manipulate information in short-term memory [18].

The CBTT evaluates visuospatial and visuoconstructive abilities. Participants are shown a sequence of blocks on a board and must tap the same sequence in the correct order. This test measures the individual’s ability to remember and reproduce spatial patterns [18].

The BSRT assesses verbal episodic memory. Participants listen to a short story and are then asked to recall as many details as possible immediately or after a delay. It evaluates the individual’s ability to retain and retrieve information from verbal narratives [19].

The PVF measures verbal fluency and executive functions. Participants are given a specific letter of the alphabet and must generate as many words as possible which start with that letter within a specified time limit. It assesses the individual’s ability to access and produce words based on phonemic rules [19].

The SVF is similar to phonemic verbal fluency, but focuses on semantic knowledge. Participants are asked to generate as many words as possible from a specific category (e.g., animals, fruits) within a given time limit. It assesses the individual’s ability to access and produce words based on semantic associations [19].

The DODT evaluates language and semantic knowledge. Participants are presented with pictures or objects and asked to name them. It assesses the individual’s ability to retrieve and produce correct names for familiar objects [20]. Additionally, the test consists of two subtests: one for figure naming, and the other for figure indication.

The SDT assesses visual perception and attention. Participants are presented with pairs of visual stimuli and must determine whether they are the same or different. It measures the individual’s ability to discriminate between visual features [21].

The CDT evaluates visuospatial and executive functions. Participants are asked to draw a clock face and set the hands to a specific time. It assesses the individual’s ability to perceive and reproduce spatial relationships and execute planning and organization [22].

The AMT measures attention and working memory. Participants are presented with a matrix of letters or numbers and must indicate whether a target stimulus is present. It assesses the individual’s ability to sustain attention and detect relevant stimuli [21].

The FAB is a brief screening tool for evaluating frontal lobe functions. It consists of several subtests that assess cognitive flexibility, motor programming, sensitivity to interference, inhibitory control, and environmental autonomy. The FAB helps identify deficits in executive functions associated with frontal lobe dysfunction [23].

Cognitive test results were recorded as raw scores for each patient. For the study, the scores were adjusted for age and education, and equivalent scores were considered (0 = deficit, 1 = borderline, 2, 3, 4 = normal). This paper focuses on the cognitive outcome variables. Our objective is to analyze the results of the cognitive tests administered to patients before (T0) and 10 days after the intervention (T1) to identify any deficits related to the cognitive functions assessed using each test mentioned above. The variations observed between the results of the pre-intervention and post-intervention tests will be evaluated to monitor the patients’ cognitive abilities across different time periods.

The study was fully financed under the Italian Ministry of Health project (RF-2016-02361069) titled “Predicting cerebrovascular events after aortic valve replacement procedures through the assessment of preprocedural cardiovascular and cerebrovascular control indexes (PEARL)”. It was reviewed and approved by the Ethics Committee of San Raffaele Hospital in Milan, Italy, on 5 April 2018 (registration number 68/int/2018). The study was conducted in accordance with the principles stated in the Declaration of Helsinki, Good Clinical Practice (GCP), institutional regulations, and Italian laws and guidelines. Written informed consent was obtained from all patients.

The study involved the enrollment of 104 adult patients who were admitted to the IRCCS Policlinico San Donato for surgical aortic valve replacement (SAVR). The study duration was 36 months, starting from 1 June 2018. The first patient was enrolled on 8 October 2018.

Inclusion criteria:

- Age ≥ 18 years.
- Indication for aortic valve replacement surgery via SAVR.
- Presence of spontaneous sinus rhythm.
- Signed informed consent.
- Exclusion criteria:
- Age less than 18 years.
- Absence of spontaneous sinus rhythm.
- Inability to sign informed consent.
- Pregnancy.

## 7. Statistical Analysis

Numerical variables are reported as mean ± standard deviation and median (IQR), while categorical variables are reported as count and percentage. The normality of continuous variables was assessed using the Shapiro–Wilk test. T0 and T1 scores were compared with a non-parametric Wilcoxon matched-pairs signed-rank tests. We kept a *p* < 0.05 significance level. The statistical analysis was conducted using SAS version 9.4 (SAS Institute, Cary, NC, USA).

## 8. Results

A total of 104 patients were eligible for the study; however, only 99 were assessed at the initial time point (T0) prior to surgery. The reduction in sample size at T0 was due to several factors: some patients initially signed informed consent but later withdrew, citing concerns about the upcoming surgery, while others were not interested in participating in the trial. Moreover, the limited time between hospital admission and surgery introduced logistical challenges, reducing the likelihood of completing the T0 assessment. Specific barriers included patients undergoing preoperative procedures, such as coronary angiography or other diagnostic exams, which interfered with the scheduling of pre-surgical cognitive assessments.

At the second time point (T1), following surgery, only 64 of the 99 patients assessed at T0 completed the evaluation. This decline in patient participation was primarily attributed to post-surgical complications and additional logistical challenges. Several patients required further surgical interventions due to complications, making them unavailable for the follow-up assessment at T1. Others were physically unable to perform the required post-operative tests due to health limitations, such as prolonged recovery or medical restrictions. Furthermore, some patients were discharged early to cardiac rehabilitation centers before the planned 7-day post-surgery assessment, preventing their participation in the T1 evaluation. These factors contributed to the reduction in follow-up data available at T1.

As a result, it was decided to proceed with statistical analysis exclusively on a subset of 64 SAVR patients who completed all tests at both T0 and T1 to obtain a meaningful comparison. The evaluated subset consisted of 43 males and 21 females, with a mean age of 61.47 ± 13.78 years, ranging from 25 to 82 years. Of this sample, 15.6% (*n* = 10) had completed primary school, 35.9% (*n* = 23) had completed lower secondary school, 23.4% (*n* = 15) had completed upper secondary school, 10.9% (*n* = 7) held a bachelor’s degree, and 14.1% (*n* = 9) held a master’s degree. Therefore, the statistical analysis was conducted on this subset.

Mean ± std, median (25th percentile–75th percentile).

Regarding Table 1 with a Bonferroni correction, only one parameter remained statistically significant, the Babcock Story Recall Test (BSRT), with a *p*-value of 0.0002.

To assess and compare the results between T0 and T1, the researchers applied scores adjusted for age and education, where applicable. In cases where score adjustment was not available, a threshold value was used.

For cognitive screening, the Mini Mental State Examination (MMSE) was administered. At T0, 96.9% of the population (*n* = 62 patients) obtained a score within the normal range, 1.6% (*n* = 1 patient) achieved a borderline score, and 1.6% (*n* = 1 patient) obtained a deficient score. The average MMSE score at T0 was 28.50 ± 1.79.

**Figure 1:**
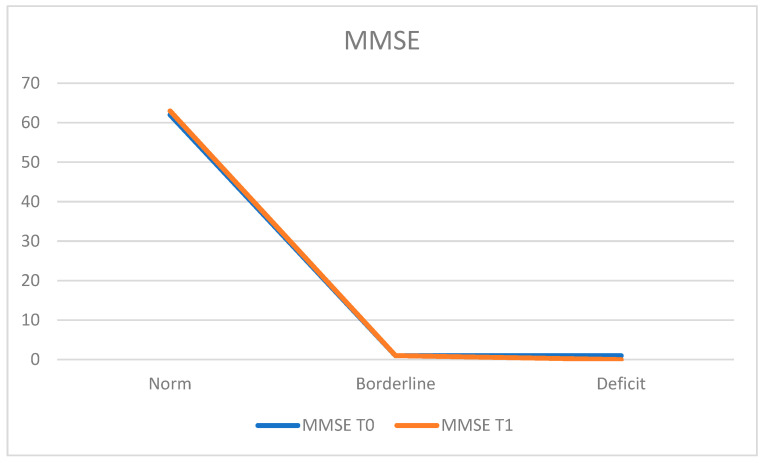
Mini Mental State Examination (MMSE).

At the T1 retest, 98.4% of the population (*n* = 63 patients) scored within the normal range, while 1.6% (*n* = 1 patient) achieved a borderline score. The average MMSE score at T1 was 28.61 ± 1.61 (Figure 1).

For executive function screening, the Frontal Assessment Battery (FAB) was administered.

At T0, 54.7% of the population (*n* = 35 patients) obtained normal executive functions, while 29.7% (*n* = 19 patients) displayed deficient scores and 15.6% (*n* = 10 patients) achieved borderline scores. The average FAB score at T0 was 14.47 ± 2.86.

**Figure 2:**
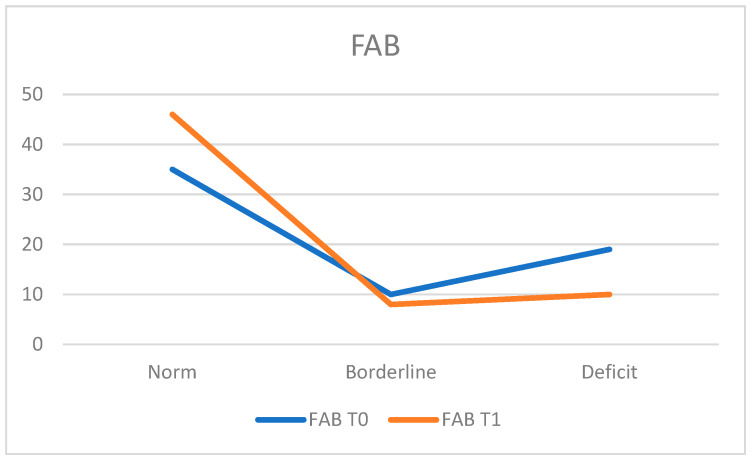
Frontal Assessment Battery (FAB).

At T1, 71.9% (*n* = 49 patients) of the population fell within the normal range, while 15.6% (*n* = 10 patients) still presented deficits and 12.5% (*n* = 8 patients) obtained borderline scores. The average FAB score at T1 was 15.15 ± 2.60, indicating an improvement in executive performance at T1, considering that the number of previously deficient patients decreased from 19 at T0 to 10 at T1 (Figure 2).

For the assessment of attentional skills, the Attentive Matrices Test (AMT) was utilized.

At T0, 95.3% of the population (*n* = 61 patients) obtained scores within the normal range, while 3.1% (*n* = 2 patients) demonstrated deficient performance, and 1.6% (*n* = 1 patient) achieved a borderline score. The average score was 47.75 ± 7.08.

**Figure 3:**
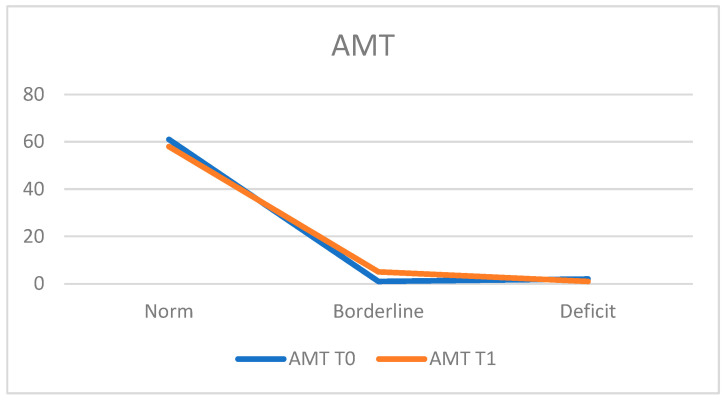
Attentive Matrices Test (AMT).

At T1, 90.6% (*n* = 58 patients) achieved scores within the normal range, 1.6% (*n* = 1 patient) exhibited deficits, and 7.8% (*n* = 5 patients) obtained a borderline score. The average score at T1 was 46.65 ± 7.99, indicating a slight decline in performance (Figure 3).

In the assessment of attentive and visual perception using the Segments Discrimination Test (SDT), at T0, 98.4% of the population (*n* = 63 patients) demonstrated normal functioning, while 1.6% (*n* = 1 patient) achieved a borderline score.

The average score was 27.84 ± 2.76.

At T1, 96.8% (*n* = 62 patients) remained within the normal range, 1.6% (*n* = 1 patient) exhibited deficits, and 1.6% (*n* = 1 patient) obtained a borderline score.

**Figure 4:**
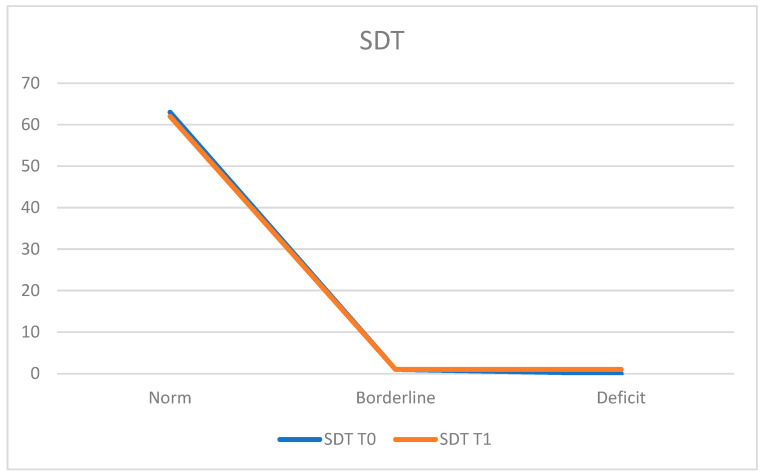
Segments Discrimination Test (SDT).

The average score at T1 was 27.47 ± 2.82 (Figure 4).

In the assessment of language and semantic knowledge using the “Denomination and Objects Detection” (DODT) test, the entire population was found to be within the normal range at both T0 and T1 across both trials.

The average score at T0 was 14.23 ± 1.15, and at T1 it was 14.63 ± 0.83.

To assess short-term verbal memory and working memory using the Digit Span Test (DST), at T0, 93.8% of the population (*n* = 60 patients) demonstrated normal functioning, while 3.1% (*n* = 2 patients) obtained deficient scores and 3.1% (*n* = 2 patients) achieved borderline scores.

The average score at T0 was 5.74 ± 1.26.

At T1, 93.8% (*n* = 60 patients) remained within the normal range, 1.6% (*n* = 1 patient) exhibited deficits, and 4.7% (*n* = 3 patients) obtained borderline scores.

**Figure 5:**
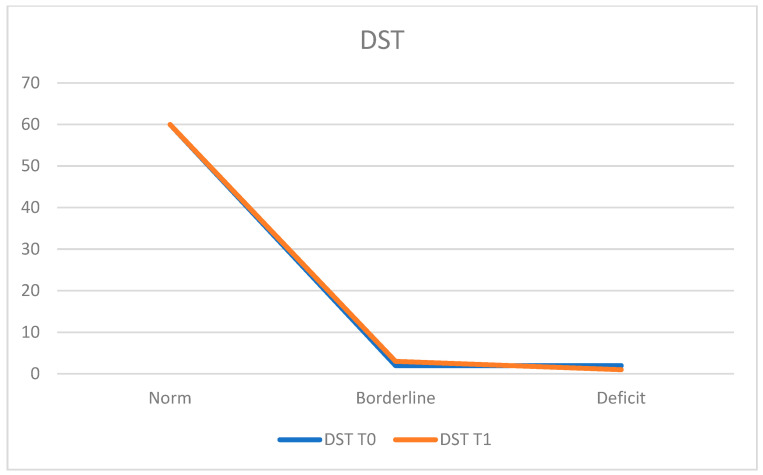
Denomination and Objects Detection (DODT).

The average score at T1 was 5.83 ± 1.20 (Figure 5).

To assess visuospatial and visuoconstructive skills, the Corsi Block Tapping Test (CBTT) was administered. At T0, 95.3% of the population (*n* = 61 patients) demonstrated normal functioning, while 3.1% (*n* = 2 patients) exhibited deficits and 1.6% (*n* = 1 patient) achieved a borderline score. The average score was 5.18 ± 0.86.

**Figure 6:**
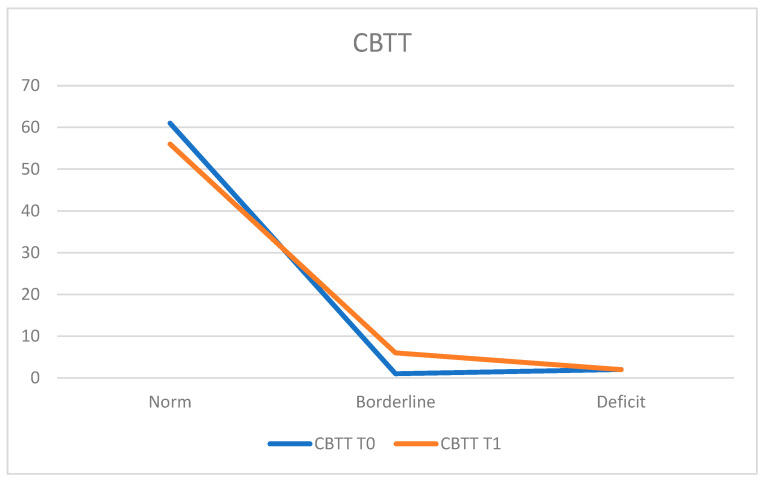
Corsi Block Tapping Test (CBTT).

At T1, 87.5% (*n* = 56 patients) fell within the normal range, 3.1% (*n* = 2 patients) showed deficits, and 9.4% (*n* = 6 patients) obtained a borderline score. The average score at T1 was 4.97 ± 0.86. A slight decline in performance is noted, considering that three patients who scored within the normal range at T0 did not achieve the same performance at T1, moving to a borderline score (Figure 6).

To assess verbal episodic memory, the Babcock Story Recall Test (BSRT) was administered. At T0, 73.4% of the population (*n* = 47 patients) obtained results within the normal range, while 10.9% (*n* = 7 patients) exhibited deficits and 15.6% (*n* = 10 patients) achieved borderline scores.

The average score was 13.43 ± 4.08.

At T1, 92.2% (*n* = 59 patients) fell within the normal range, 1.6% (*n* = 1 patient) displayed deficits, and 6.3% (*n* = 4 patients) obtained borderline scores.

**Figure 7:**
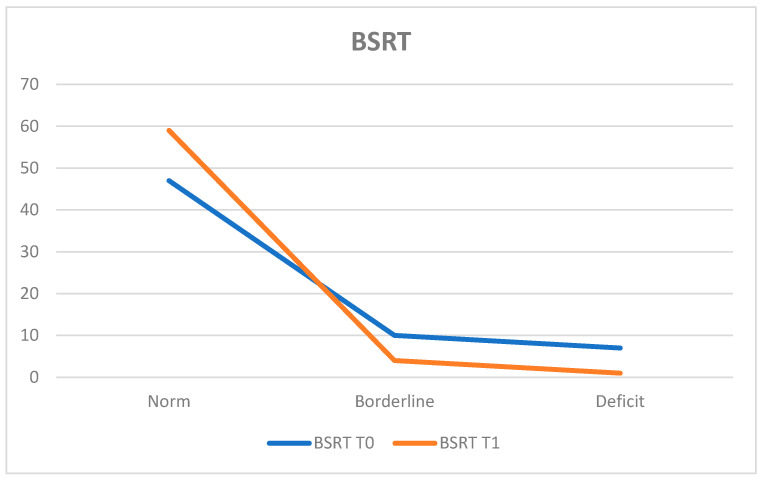
Babcock Story Recall Test (BSRT).

The average score at T1 was 17.16 ± 4.88, with a statistically significant difference between T0 and T1 (*p* < 0.0001). Notably, there was an improvement among patients who had deficit scores at T0, as the number of such patients decreased by six units by T1 (Figure 7).

The Clock Drawing Test (CDT) assessed visuospatial and executive functions. At T0, 78.1% of the population (*n* = 50 patients) obtained results within the normal range, while 12.5% (*n* = 8 patients) exhibited deficits and 9.4% (*n* = 6 patients) achieved borderline scores.

The average score was 52.24 ± 9.48.

At T1, 84.4% (*n* = 54 patients) remained within the normal range, 6.3% (*n* = 4 patients) presented deficits, and 9.4% (*n* = 6 patients) obtained borderline scores.

**Figure 8:**
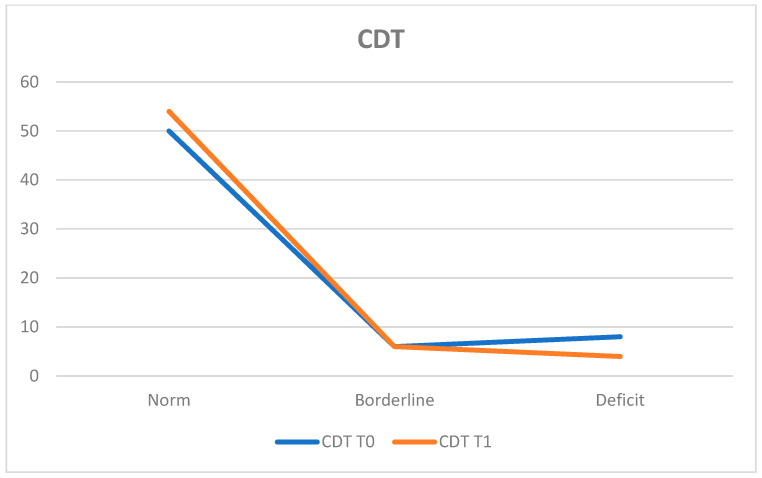
Clock Drawing Test (CDT).

The average score at T1 was 53.16 ± 11.15. A slight improvement in performance is noted, especially among patients who had deficit scores at T0, as the number of patients with deficits decreased from eight to four by T1 (Figure 8).

The Phonemic Verbal Fluency Test (PVF) assessed phonemic word production. At T0, 90.6% of the population (*n* = 58 patients) obtained results within the normal range, while 4.7% (*n* = 3 patients) exhibited deficits and 4.7% (*n* = 3 patients) achieved borderline scores.

The average score was 36.34 ± 10.93.

At T1, 90.6% (*n* = 58 patients) remained within the normal range, 1.6% (*n* = 1 patient) showed deficient performance, and 7.8% (*n* = 5 patients) obtained borderline scores.

The average score at T1 was 37.05 ± 10.47.

**Figure 9:**
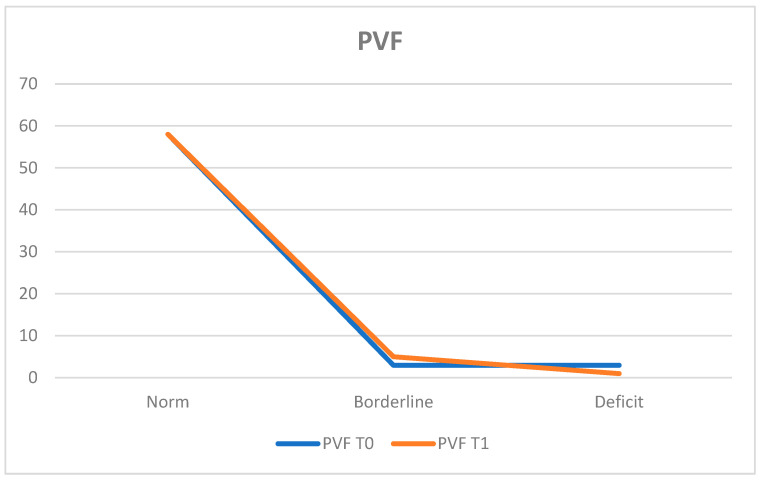
Verbal Fluency Test (PVF).

These results indicate a stable condition between T0 and T1 (Figure 9).

The Semantic Verbal Fluency Test (SVF) assessed semantic knowledge. At T0, 92.2% of the population (*n* = 59 patients) obtained results within the normal range, while 4.7% (*n* = 3 patients) exhibited deficits and 3.1% (*n* = 2 patients) achieved borderline scores.

The average score was 44.40 ± 10.49.

At T1, 90.6% (*n* = 58 patients) remained within the normal range, 3.1% (*n* = 2 patients) showed deficits, and 6.3% (*n* = 4 patients) obtained borderline scores.

**Figure 10:**
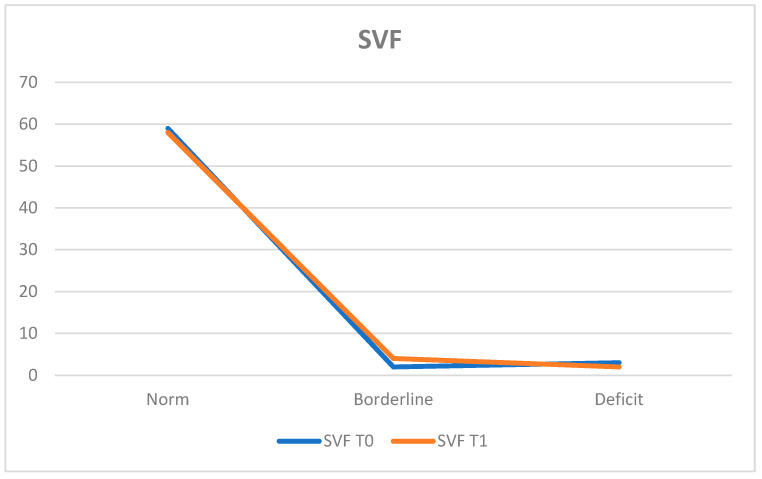
Semantic Verbal Fluency Test (SVF).

The average score at T1 was 45.16 ± 11.70 (Figure 10).

## 9. Discussion and Conclusions

Aortic valve stenosis (AVS) is a common valvular heart disease that has significant implications for neurocognitive and neuropsychological functioning, ultimately affecting patients’ quality of life. This study aimed to evaluate the neurocognitive effects of surgical aortic valve replacement (SAVR) on patients with aortic stenosis who underwent the procedure at IRCCS Policlinico San Donato. Specifically, we sought to examine the association between SAVR and cognitive outcomes by utilizing a comprehensive battery of neuropsychological and cognitive tests administered before and after surgery.

Our results indicate that SAVR does not significantly alter overall cognitive function in patients with aortic stenosis, except for notable improvements in verbal episodic memory as assessed using the Babcock Story Recall Test (BSRT). The data showed a statistically significant increase in the percentage of patients scoring within the normal range on the BSRT, rising from 73.4% at baseline (T0) to 92.2% post-surgery (T1). The average BSRT score also improved significantly from 13.43 ± 4.08 at T0 to 17.16 ± 4.88 at T1 (*p* < 0.0001). Additionally, the number of patients with deficit scores decreased, suggesting a substantial cognitive benefit of SAVR in this domain.

The observed improvement in verbal episodic memory following SAVR aligns with previous studies suggesting cognitive benefits associated with cardiac surgery, particularly in the domains of memory and executive functions [24,25,26].

In our study, the Mini Mental State Examination (MMSE) indicated high baseline cognitive functioning, with 96.9% of patients scoring within the normal range at T0, which increased to 98.4% at T1, showing a slight improvement in average scores (28.50 ± 1.79 to 28.61 ± 1.61). Furthermore, executive functions, evaluated using the Frontal Assessment Battery (FAB), demonstrated a significant increase in the percentage of patients with normal scores from 54.7% at T0 to 71.9% at T1, along with an increase in the average FAB score from 14.47 ± 2.86 to 15.15 ± 2.60.

However, we also noted a decline in some cognitive domains, particularly in attentional skills measured using the Attentive Matrices Test (AMT), where the proportion of patients scoring within the normal range decreased from 95.3% at T0 to 90.6% at T1, along with a slight drop in average scores (47.75 ± 7.08 to 46.65 ± 7.99). Additionally, visuospatial and visuoconstructive skills, assessed via the Corsi Block Tapping Test (CBTT), showed a decline in normal functioning from 95.3% at T0 to 87.5% at T1, with an average score decrease from 5.18 ± 0.86 to 4.97 ± 0.86. This decline is concerning, especially since three patients who performed normally at T0 moved to a borderline score at T1.

Further evaluations using the Clock Drawing Test (CDT) and the Phonemic and Semantic Verbal Fluency Tests (PVF and SVF) revealed mixed results. The CDT indicated stability in visuospatial and executive functions, with a slight increase in the percentage of patients within the normal range from 78.1% at T0 to 84.4% at T1, despite a decrease in average scores (52.24 ± 9.48 to 53.16 ± 11.15). Both the PVF and SVF demonstrated stability, with the former maintaining 90.6% normal functioning at both time points and a slight average score increase (36.34 ± 10.93 to 37.05 ± 10.47). Similarly, the SVF indicated stability with a decrease in the normal range from 92.2% to 90.6%, but the average score remained comparable (44.40 ± 10.49 to 45.16 ± 11.70).

These findings suggest that while SAVR provides significant cognitive benefits in verbal episodic memory and some aspects of executive functioning, challenges remain in attentional and visuospatial domains. The observed decline in these areas may reflect the complexity of cognitive recovery post-surgery and highlight the necessity for targeted interventions.

It is crucial to note that this study faced limitations regarding sample size and participant retention. A total of 104 patients were initially eligible, but only 99 were assessed at T0. By T1, only 64 patients completed evaluations, primarily due to post-surgical complications and logistical challenges surrounding patient availability for follow-up assessments. The decision to focus statistical analysis on the 64 patients who completed both assessments provides a more reliable evaluation of cognitive outcomes, yet it limits the generalizability of our findings.

While this study focuses on patients undergoing SAVR, it is important to consider the natural history of aortic stenosis in patients who do not undergo surgical correction. In the absence of intervention, the progression of aortic stenosis is often characterized by worsening symptoms such as dyspnea, angina, and syncope, ultimately leading to a decline in functional capacity and quality of life. Without surgical intervention, these patients are also at increased risk of heart failure, arrhythmias, and sudden cardiac death. Additionally, cognitive decline may occur as a result of chronic cerebral hypoperfusion caused by the compromised cardiac output associated with advanced aortic stenosis.

In light of our results, we recommend incorporating cognitive evaluation as a critical endpoint when assessing the benefits of therapeutic interventions aimed at improving cerebral perfusion in patients with aortic stenosis. Early cognitive screening may help identify subtle deficits that could otherwise remain unnoticed, allowing for timely interventions and enhancing the overall understanding of treatment impacts. Moreover, the development of tailored cognitive rehabilitation programs, alongside surgical or pharmacological treatments, could significantly enhance long-term outcomes by addressing cognitive impairments and promoting recovery, ultimately improving patients’ quality of life.

In conclusion, while SAVR offers cognitive benefits, particularly in verbal episodic memory and executive functions, the challenges in patient recruitment and retention highlight the need for improved strategies to assess cognitive outcomes in this population. Future research should explore the potential benefits of integrating routine cognitive assessments and rehabilitation protocols into standard care to optimize therapeutic success for patients undergoing interventions for aortic stenosis.

## 10. Limitations

First and foremost, one of the primary limitations of this study is its small sample size, which limits the ability to generalize the results to a broader population. Additionally, the relatively short follow-up period may restrict the long-term applicability of our findings.

A significant limitation of this study is the lack of a control group. Without a comparison group of patients undergoing alternative treatments, such as transcatheter aortic valve replacement (TAVI) or those receiving medical management alone, we cannot directly compare cognitive outcomes across different treatment approaches. This absence of a control group constrains our ability to assess the relative effectiveness of SAVR compared to other interventions.

Moreover, this study encountered challenges in obtaining data from TAVI patients. TAVI, typically performed on high-risk elderly patients, is less frequently performed than SAVR and often involves patients with complex health conditions that may impact their willingness or ability to participate in studies. This limitation highlights the need for future research to include diverse treatment modalities and larger patient cohorts to provide a more comprehensive understanding of cognitive outcomes.

It is also important to acknowledge that patients’ responses to cognitive tests may have been influenced by their psychological state, such as anxiety or depression related to the surgical procedure. While these factors could potentially affect cognitive performance, they were not specifically measured or included in the statistical analysis.

Finally, confounding factors such as age, pre-existing cognitive impairment, comorbidities, and perioperative complications could impact cognitive function independently of the surgical intervention. Future studies should aim to include larger sample sizes and control for these variables to better elucidate the relationship between SAVR and cognitive outcomes.

In conclusion, while this study provides evidence that SAVR is not significantly associated with cognitive decline in patients with AS, except for improvements in verbal episodic memory, the limitations outlined underscore the need for further research with more comprehensive designs and longer follow-up periods to validate and expand upon these findings.

## Figures and Tables

**Table 1:** Analysis of scores.

	T0 (N = 64)		T1 (N = 64)		*p*-Value	Cut-Off
	N		N			
MMSE	64	28.50 ± 1.79, 29.12 (27.46–30.00)	64	28.61 ± 1.61, 29.20 (27.48–30.00)	0.96	23.80
FAB	64	14.47 ± 2.86, 14.80 (12.90–16.75)	64	15.15 ± 2.60, 15.80 (14.20–16.90)	0.13	13.50
AMT	64	47.75 ± 7.08, 47.38 (43.25–53.50)	64	46.65 ± 7.99, 46.75 (40.75–52.25)	0.40	30
SDT	64	27.84 ± 2.76, 28.00 (26.00–30.00)	64	27.47 ± 2.82, 28.00 (26.00–29.50)	0.54	18
DODT denomination	64	14.23 ± 1.15, 15.00 (14.00–15.00)	64	14.63 ± 0.83, 15.00 (15.00–15.00)	0.02	10
DODT detection	64	23.91 ± 0.34, 24.00 (24.00–24.00)	64	23.92 ± 0.32, 24.00 (24.00–24.00)	0.98	22
DST	64	5.74 ± 1.26, 5.75 (4.75–6.50)	64	5.83 ± 1.20, 5.75 (5.25–6.50)	0.78	3.75
CBTT	64	5.18 ± 0.86, 5.25 (4.50–5.75)	64	4.97 ± 0.86, 5.13 (4.38–5.50)	0.17	3.5
BSRT	64	13.43 ± 4.08, 13.75 (10.00–16.50)	64	17.16 ± 4.88, 17.00 (13.50–20.75)	<0.0001	8
CDT	64	52.24 ± 9.48, 54.75 (48.00–60.00)	64	53.16 ± 11.15, 56.25 (51.75–60.00)	0.29	42.17
PVF	64	36.34 ± 10.93, 36.50 (29.00–44.00)	64	37.05 ± 10.47, 37.00 (31.00–40.50)	0.82	17
SVF	64	44.40 ± 10.49, 45.00 (37.50–51.50)	64	45.16 ± 11.70, 44.00 (37.00–52.50)	0.97	25

## Data Availability

The datasets presented in this article are not readily available due to participant privacy considerations. Data sharing is restricted by privacy and consent limitations; therefore, the data cannot be made publicly accessible. However, researchers interested in accessing the datasets for academic purposes may submit a request to the Clinical Psychology Service of IRCCS Policlinico San Donato at the following email address: psicologia.psd@grupposandonato.it.
